# U-shaped pattern in the association between serum magnesium levels and long-term dementia risk after traumatic brain injury: a retrospective cohort study

**DOI:** 10.3389/fnut.2026.1870929

**Published:** 2026-08-07

**Authors:** Kuo-Chuan Hung, Hsiu-Lan Weng, Yi-Chen Lai, I-Wen Chen

**Affiliations:** 1 Department of Anesthesiology, Chi Mei Medical Center, Tainan City, Taiwan; 2 Department of Anesthesiology, E-Da Hospital, I-Shou University, Kaohsiung City, Taiwan; 3 Department of Anesthesiology, Chi Mei Hospital, Liouying, Tainan City, Taiwan

**Keywords:** dementia, magnesium, propensity score matching, traumatic brain injury, TriNetX, U-shaped association

## Abstract

**Background:**

In the general population, abnormal serum magnesium levels are associated with an increased risk of cognitive decline and dementia. However, whether this association extends to patients with traumatic brain injury (TBI), a population with distinct neurobiological vulnerabilities, remains unknown.

**Methods:**

Using the TriNetX Global Collaborative Network, we identified adults aged ≥50 years with a history of intracranial injury (ICD-10-CM: S06). Patients with repeatedly low serum magnesium levels (< 1.7 mg/dL, two measurements within 1 year) were compared with those with normal levels (1.70–2.20 mg/dL) using 1:1 propensity score matching. A 1-year landmark period was applied to reduce the reverse causation. The primary outcome was incident dementia over 10 years of follow-up. The secondary outcomes included dementia subtypes, cognitive dysfunction, and all-cause mortality. To explore a potential U-shaped relationship, a parallel analysis was conducted comparing patients with repeatedly high magnesium levels (>2.20 mg/dL) with the same reference cohort. Sensitivity analyses, subgroup analyses by sex and TBI severity, and multivariable Cox regression were performed.

**Results:**

After matching, 11,716 patients were retained in each cohort. Low magnesium levels were associated with a higher 10-year risk of incident dementia [2.53% vs. 1.78%; hazard ratio (HR) 1.62, 95% confidence interval (CI), 1.36–1.94; *p* < 0.001]. Among the secondary outcomes, associations were observed for vascular dementia (HR 1.97, *p* = 0.001), other dementia types (HR 1.63, *p* < 0.001), cognitive dysfunction (HR 1.43, *p* = 0.009), and all-cause mortality (HR 1.29, *p* < 0.001), whereas Alzheimer's disease did not reach significance (HR 1.43, *p* = 0.075). High magnesium levels were similarly associated with an increased risk of dementia (HR 1.57, *p* < 0.001). The findings were consistent across sensitivity and subgroup analyses and after multivariable adjustment (adjusted HR 1.50, *p* < 0.001).

**Conclusion:**

In adults with prior TBI, both low and high serum magnesium levels were associated with a greater long-term risk of dementia, suggesting a U-shaped relationship. Prospective studies are needed to determine whether monitoring or modifying magnesium status can alter the post-traumatic dementia trajectory.

## 1 Introduction

Traumatic brain injury (TBI) is a major cause of long-term neurological disability and has been consistently associated with an increased risk of subsequent dementia (1–4). However, the biological and clinical factors that determine which TBI survivors progress to dementia remain incompletely understood (4–6). The mechanisms linking TBI to later neurodegeneration are multifactorial and include chronic neuroinflammation, progressive axonal injury, vascular dysfunction, oxidative stress, and calcium-mediated excitotoxicity (7–12), which may persist long after the initial injuries. Given the growing number of older adults surviving TBI, the identification of clinically measurable factors associated with post-traumatic dementia risk is of increasing importance.

Magnesium is essential for neuronal and vascular homeostasis, contributing to neurotransmission, NMDA receptor regulation, neuroinflammatory modulation, and endothelial barrier and vascular function (13–16). It modulates N-methyl-D-aspartate receptor activity, calcium influx, synaptic function, oxidative stress, and inflammatory signaling (14, 17, 18). These pathways are also central to secondary injury and neurodegenerative processes after TBI (19, 20), suggesting that magnesium dysregulation may be particularly relevant in patients with a prior brain injury. In general adult populations, abnormal serum magnesium levels have been associated with cognitive decline and dementia (21–26), and emerging evidence suggests that this relationship may be non-linear, with both low and high magnesium levels potentially reflecting adverse biological states (23, 25).

However, existing evidence has largely been derived from general population cohorts (21, 23–25), and longitudinal data specifically addressing patients with TBI are limited. This represents an important knowledge gap because patients with prior TBI may have distinct cognitive trajectories and may be vulnerable to magnesium dysregulation owing to posttraumatic physiological changes, comorbid illnesses, medication exposure, hospitalization-related factors, and alcohol-related disorders. Whether magnesium abnormalities are independently associated with incident dementia in this high-risk population remains to be elucidated. Therefore, we conducted a propensity score–matched retrospective cohort study using the TriNetX Global Collaborative Network to examine the association between low serum magnesium status and the 10-year risk of incident dementia among adults with a history of TBI. High magnesium status was also evaluated in a parallel analysis to explore whether abnormal magnesium levels were associated with the risk of dementia in both directions.

## 2 Methods

### 2.1 Data source and ethical statement

This retrospective propensity score–matched cohort study used de-identified electronic health record data from the TriNetX Global Collaborative Network, a federated research platform comprising 173 healthcare organizations. The TriNetX platform has been increasingly used in real-world observational research across multiple clinical fields (27–30). Because the TriNetX database provides only aggregated, de-identified data, the requirement for informed consent was waived. The study protocol was approved by the Institutional Review Board of Chi Mei Medical Center, Tainan, Taiwan. All study procedures were conducted in accordance with the ethical principles of the Declaration of Helsinki and the relevant institutional guidelines. This study was reported in accordance with the Strengthening the Reporting of Observational Studies in Epidemiology (STROBE) guideline for cohort studies.

### 2.2 Exposure definition

The study population included patients aged ≥50 years with a documented history of intracranial injury (ICD-10-CM code: S06) between January 1, 2010, and December 31, 2023. To minimize the influence of acute posttraumatic physiological changes, TBI diagnosis was required to have occurred at least 3 months before the index date. Patients were categorized according to their serum magnesium status based on repeated measurements. The magnesium deficiency cohort included patients with repeatedly low serum magnesium levels, defined as two qualifying measurements <1.7 mg/dL within 1 year. The reference cohort included patients with repeatedly normal serum magnesium levels, defined as two qualifying measurements between 1.70 and 2.20 mg/dL within 1 year. The index date was defined as the date of the second qualifying magnesium measurement, thereby ensuring that the magnesium status was established before the start of follow-up. This repeated-measurement criterion was selected to reduce exposure misclassification from isolated or transient electrolyte abnormalities. The 1-year window was intended to balance the identification of a sustained magnesium pattern with preservation of cohort size and clinical applicability. Therefore, magnesium status in this study should be interpreted as repeatedly documented low, normal, or high serum magnesium within a clinically relevant assessment period, rather than as a direct measure of total-body magnesium stores.

### 2.3 Exclusion criteria

Patients with pre-existing dementia-related diagnoses before the index date were excluded to establish an incident dementia cohort. These included vascular dementia (F01), dementia in other diseases classified elsewhere (F02), unspecified dementia (F03), and other degenerative nervous system diseases (G30–G32). Patients were also excluded if they had conditions that could substantially confound magnesium status, dementia risk, or outcome ascertainment, including end-stage renal disease (N18.6), chronic kidney disease stage 4 or 5 (N18.4, N18.5), dependence on renal dialysis (Z99.2), bipolar disorder (F31), schizophrenia spectrum or other non-mood psychotic disorders (F20–F29), Parkinson's disease (G20), and prior bariatric surgery (Z98.84; CPT: 1007385). Patients with cerebral infarction (I63) or non-traumatic intracerebral hemorrhage (I61) within 1 year before the index date were excluded to minimize confounding factors from non-traumatic cerebrovascular causes of cognitive decline. To reduce the risk of reverse causation, a 1-year landmark period was applied, and patients diagnosed with dementia or other degenerative diseases of the nervous system within 1 year of the index date were excluded.

### 2.4 Data collection and propensity score matching

To minimize confounding between the magnesium deficiency and reference cohorts, 1:1 propensity score matching was performed using a greedy nearest-neighbor algorithm with a caliper width of 0.1 pooled standard deviations. This caliper-based nearest-neighbor matching approach has been widely used in observational propensity score analyses to reduce inappropriate matches and improve covariate balance, and the same 0.1 pooled standard-deviation caliper has been applied in multiple TriNetX-based cohort studies (31–36). The propensity score model included variables considered clinically relevant to both magnesium status and dementia risk, with emphasis on demographic characteristics, vascular and metabolic comorbidities, neuropsychiatric conditions, TBI subtypes and severity-related diagnoses, medication exposure, and laboratory indicators reflecting renal function, systemic inflammation, nutritional status, glycemic control, anemia, and body composition. Medication covariates included drug classes that may influence magnesium homeostasis, neurological status, or cognitive outcomes. Laboratory covariates were incorporated to account for the baseline physiological reserve and potential nutritional or metabolic vulnerability. The full list of variables used for matching is presented in Supplementary Table 1. Covariate balance after matching was evaluated using standardized mean differences, with a standardized mean differences (SMDs) < 0.10 considered indicative of adequate balance.

### 2.5 Primary and secondary outcomes

The primary outcome was newly diagnosed dementia (ICD-10-CM codes: F01, F02, F03, or G30), evaluated over a 10-year follow-up period after the 1-year landmark. Secondary outcomes included dementia subtypes [i.e., Alzheimer's disease (G30), vascular dementia (F01), and other dementia types (F02, F03)], mild cognitive impairment (G31.84), and all-cause mortality, assessed over the same period. Patients were followed up until the outcome of interest, death, last recorded clinical encounter, or the end of the 10-year follow-up window, whichever occurred first. All primary and secondary outcomes were assessed at 5 years to characterize the temporal pattern of associations between low magnesium status and incident cognitive outcomes.

Additional prespecified outcomes were used to support the interpretation. Serum calcium < 8.5 mg/dL was included as a positive control outcome because of the established physiological relationship between magnesium and calcium homeostasis. Acute appendicitis (K35) was selected as a negative control outcome because no biologically plausible association with magnesium status was expected. Healthcare utilization during follow-up was evaluated by assessing hospital visits to identify potential surveillance bias. The persistence of low magnesium status was examined by comparing subsequent low magnesium events between cohorts during follow-up.

### 2.6 Sensitivity analyses, subgroup analyses, and multivariable regression

Three prespecified sensitivity analyses were performed to test the robustness of the findings. To address potential competing mortality, Model I excluded patients who died during the follow-up period. To reduce the possibility of outcome under-ascertainment, Model II included only patients with more than one healthcare encounter during the follow-up. To assess whether the association persisted in a population with a higher baseline dementia risk, Model III was restricted to patients older than 65 years.

Prespecified subgroup analyses were performed according to sex and TBI severity of TBI. TBI severity was operationalized using ICD-10-CM diagnostic patterns. Mild TBI was identified using concussion codes (S06.0), whereas moderate-to-severe TBI was defined using structural intracranial injury codes, including traumatic subdural hemorrhage (S06.5), traumatic subarachnoid hemorrhage (S06.6), epidural hemorrhage (S06.4), and other non-concussion intracranial injuries within the S06 code family. Propensity score matching and outcome analyses were repeated separately within each subgroup, and interaction terms were used to test heterogeneity.

To further evaluate the independent association between low magnesium status and incident dementia, a multivariable Cox proportional hazards regression model was constructed incorporating clinically relevant covariates, including age at index, sex, essential hypertension, diabetes mellitus, nicotine dependence, mood disorders, alcohol-related disorders, cerebrovascular diseases, liver diseases, overweight and obesity, disorders of the thyroid gland, chronic kidney disease, malnutrition, and TBI subtypes (traumatic subdural hemorrhage and traumatic subarachnoid hemorrhage).

### 2.7 Assessment of high magnesium status

To evaluate a potential U-shaped relationship between serum magnesium levels and dementia risk, a separate analysis was conducted comparing patients with repeatedly high magnesium status (>2.20 mg/dL, defined by two qualifying measurements within 1 year) with the same reference cohort of patients with normal magnesium levels (1.70–2.20 mg/dL). The same study design, exclusion criteria, propensity score matching strategy, and analytical framework applied in the primary analysis were replicated for this comparison. Primary and secondary outcomes, positive and negative control outcomes, and healthcare utilization were assessed over a 10-year follow-up period. A multivariable Cox regression model incorporating the same set of covariates was also used to evaluate the independent association between high magnesium status and incident dementia.

### 2.8 Statistical analysis

Time-to-event outcomes were analyzed using cause-specific Cox proportional hazards models and reported as hazard ratios (HRs) with 95% confidence intervals (CIs). This modeling strategy was selected because the objective was to examine the etiological association between magnesium status and subsequent dementia, rather than estimating the absolute cumulative incidence while accounting for competing mortality. The proportional hazards assumption was evaluated using the Schoenfeld residuals. Dementia-free survival was illustrated using Kaplan–Meier curves and compared between groups using log-rank tests.

For the primary outcome, E-values were calculated to estimate the minimum magnitude of association that an unmeasured confounder would need to have with both low magnesium status and dementia to fully account for the observed association between them. Statistical significance for the primary outcome was defined using a two-sided α-level of 0.05. Secondary outcomes, control outcomes, sensitivity analyses, and subgroup analyses were interpreted as exploratory; therefore, no adjustments for multiple comparisons were performed. All analyses were conducted using the available data without imputation for missing values.

## 3 Results

### 3.1 Patient selection and baseline characteristics

A total of 11,805 patients with low serum magnesium and 40,457 patients with normal magnesium levels were initially identified after applying the eligibility criteria. Before matching, several baseline characteristics differed substantially between the two cohorts, particularly the prevalence of hypertension, diabetes mellitus, alcohol-related disorders, liver diseases, malnutrition, and multiple medication classes, with several covariates showing SMDs above 0.10. After 1:1 propensity score matching, 11,716 patients were retained in each cohort, and all measured covariates achieved adequate balance with absolute standardized mean differences (SMDs) < 0.10 (Table 1).

**Table 1:** Baseline characteristics of patients with low magnesium status and matched controls before and after propensity score matching.

Variables	Before matching			After matching		
	Low Mg group (*n* = 11,805)	Control group (*n* = 40,457)	SMD	Low Mg group *n* = 11,716)	Control group (*n* = 11,716)	SMD
Patient characteristics						
Age at index (years)	63.1 ± 12.4	63.3 ± 13.1	0.020	63.1 ± 12.4	63.0 ± 12.7	0.010
BMI ≥30 (kg/m^2^)	3,723 (31.5)	1,1955 (29.6)	0.043	3,691 (31.5)	3,577 (30.5)	0.021
Female	5,161 (43.7)	17,754 (43.9)	0.003	5,137 (43.8)	5,165 (44.1)	0.005
White	7,986 (67.6)	27,846 (68.8)	0.025	7,937 (67.7)	7,955 (67.9)	0.003
Black or African American	1,763 (14.9)	5,252 (13.0)	0.056	1,738 (14.8)	1,718 (14.7)	0.005
Asian	398 (3.4)	1,718 (4.2)	0.046	398 (3.4)	383 (3.3)	0.007
Comorbidities and healthcare utilization						
Factors influencing health status and contact with health services	8,796 (74.5)	27,026 (66.8)	0.170	8,716 (74.4)	8,634 (73.7)	0.016
Essential (primary) hypertension	7,145 (60.5)	20,272 (50.1)	0.211	7,071 (60.4)	7,091 (60.5)	0.003
Diabetes mellitus	3,826 (32.4)	8,724 (21.6)	0.246	3,771 (32.2)	3,805 (32.5)	0.006
Nicotine dependence	3,150 (26.7)	7,598 (18.8)	0.189	3,095 (26.4)	3,055 (26.1)	0.008
Anxiety, dissociative, stress-related, somatoform and other nonpsychotic mental disorders	3,089 (26.2)	8,432 (20.8)	0.126	3,045 (26.0)	2,969 (25.3)	0.015
Mood disorders	3,061 (25.9)	8,010 (19.8)	0.146	3,020 (25.8)	2,992 (25.5)	0.005
Ischemic heart diseases	3029 (25.7)	8,170 (20.2)	0.130	2,983 (25.5)	2,942 (25.1)	0.008
Alcohol related disorders	3,047 (25.8)	5,485 (13.6)	0.312	2,975 (25.4)	2,986 (25.5)	0.002
Cerebrovascular diseases	2,628 (22.3)	6,561 (16.2)	0.154	2,579 (22.0)	2,574 (22.0)	0.001
Diseases of liver	2,452 (20.8)	4,360 (10.8)	0.277	2,388 (20.4)	2,298 (19.6)	0.019
Sleep disorders	2,331 (19.7)	6,668 (16.5)	0.085	2,303 (19.7)	2,252 (19.2)	0.011
Overweight and obesity	2,120 (18.0)	5,860 (14.5)	0.094	2,092 (17.9)	2,093 (17.9)	0.000
Disorders of thyroid gland	1,978 (16.8)	5902 (14.6)	0.060	1956 (16.7)	1857 (15.9)	0.023
COPD	1,929 (16.3)	4,463 (11.0)	0.155	1,896 (16.2)	1,883 (16.1)	0.003
Heart failure	1,750 (14.8)	4,149 (10.3)	0.138	1,716 (14.6)	1,712 (14.6)	0.001
Atrial fibrillation and flutter	1,709 (14.5)	4,658 (11.5)	0.088	1,690 (14.4)	1,660 (14.2)	0.007
Chronic kidney disease (CKD)	1,330 (11.3)	2,974 (7.4)	0.135	1,303 (11.1)	1,325 (11.3)	0.006
Vitamin D deficiency	1,212 (10.3)	3,512 (8.7)	0.054	1,199 (10.2)	1,153 (9.8)	0.013
Malnutrition	1,188 (10.1)	1,738 (4.3)	0.225	1,137 (9.7)	1,104 (9.4)	0.010
Epilepsy and recurrent seizures	1,053 (8.9)	2,514 (6.2)	0.102	1,028 (8.8)	995 (8.5)	0.010
COVID-19	428 (3.6)	982 (2.4)	0.070	420 (3.6)	413 (3.5)	0.003
Systemic connective tissue disorders	276 (2.3)	863 (2.1)	0.014	275 (2.3)	293 (2.5)	0.010
Type of TBI						
Concussion	2,183 (18.5)	7,214 (17.8)	0.017	2,166 (18.5)	2,160 (18.4)	0.001
Traumatic SDH	1,816 (15.4)	4,921 (12.2)	0.094	1,796 (15.3)	1,790 (15.3)	0.001
Traumatic SAH	1,011 (8.6)	2,673 (6.6)	0.074	1,000 (8.5)	957 (8.2)	0.013
Epidural hemorrhage	249 (2.1)	697 (1.7)	0.028	246 (2.1)	262 (2.2)	0.009
Laboratory data						
Hemoglobin ≥ 12 g/dL	8,853 (75.0)	28,584 (70.7)	0.098	8,776 (74.9)	8,673 (74.0)	0.020
Albumin ≥ 3.5 g/dL)	7,524 (63.7)	21,830 (54.0)	0.200	7,440 (63.5)	7,480 (63.8)	0.007
HbA1c ≥ 9%	905 (7.7)	1,639 (4.1)	0.154	883 (7.5)	906 (7.7)	0.007
eGFR ≥60 mL/min/1.73 m^2^	9,114 (77.2)	28,488 (70.4)	0.155	9,028 (77.1)	8,975 (76.6)	0.011
C-reactive protein≥ 10 mg/L	1,846 (15.6)	3,731 (9.2)	0.195	1,797 (15.3)	1,771 (15.1)	0.006
Vitamin B12 300-900 pg/mL	1,890 (16.0)	4,203 (10.4)	0.167	1,843 (15.7)	1,804 (15.4)	0.009
Medications						
Central nervous system medications	10,063 (85.2)	31,419 (77.7)	0.196	9,974 (85.1)	9,861 (84.2)	0.027
Benzodiazepine	6,642 (56.3)	17,305 (42.8)	0.272	6,558 (56.0)	6,496 (55.4)	0.011
Diuretics	4,510 (38.2)	11,152 (27.6)	0.228	4,437 (37.9)	4,474 (38.2)	0.007
Anticonvulsants	4,394 (37.2)	11,278 (27.9)	0.200	4,332 (37.0)	4,325 (36.9)	0.001
Magnesium supplementation	3,931 (33.3)	8,844 (21.9)	0.258	3,859 (32.9)	3,850 (32.9)	0.002
Insulins and analogs	3,415 (28.9)	6,899 (17.1)	0.285	3,343 (28.5)	3,357 (28.7)	0.003
Blood glucose-lowering drugs, excluding Insulins	2,285 (19.4)	4,844 (12.0)	0.204	2,252 (19.2)	2,255 (19.2)	0.001
Anticholinergics	1,553 (13.2)	3,796 (9.4)	0.120	1,527 (13.0)	1,476 (12.6)	0.013

Data are presented as *n* (%) or mean ± standard deviation (SD). Covariate balance was assessed using standardized mean differences (SMDs); an absolute SMD < 0.10 indicates adequate balance. BMI, body mass index; COPD, chronic obstructive pulmonary disease; CKD, chronic kidney disease; COVID-19, coronavirus disease 2,019; eGFR, estimated glomerular filtration rate; HbA1c, hemoglobin A1c; SDH, subdural hemorrhage; SAH, subarachnoid hemorrhage; TBI, traumatic brain injury; Mg, magnesium; HR, hazard ratio; CI, confidence interval.

### 3.2 Outcomes

**Figure 1:**
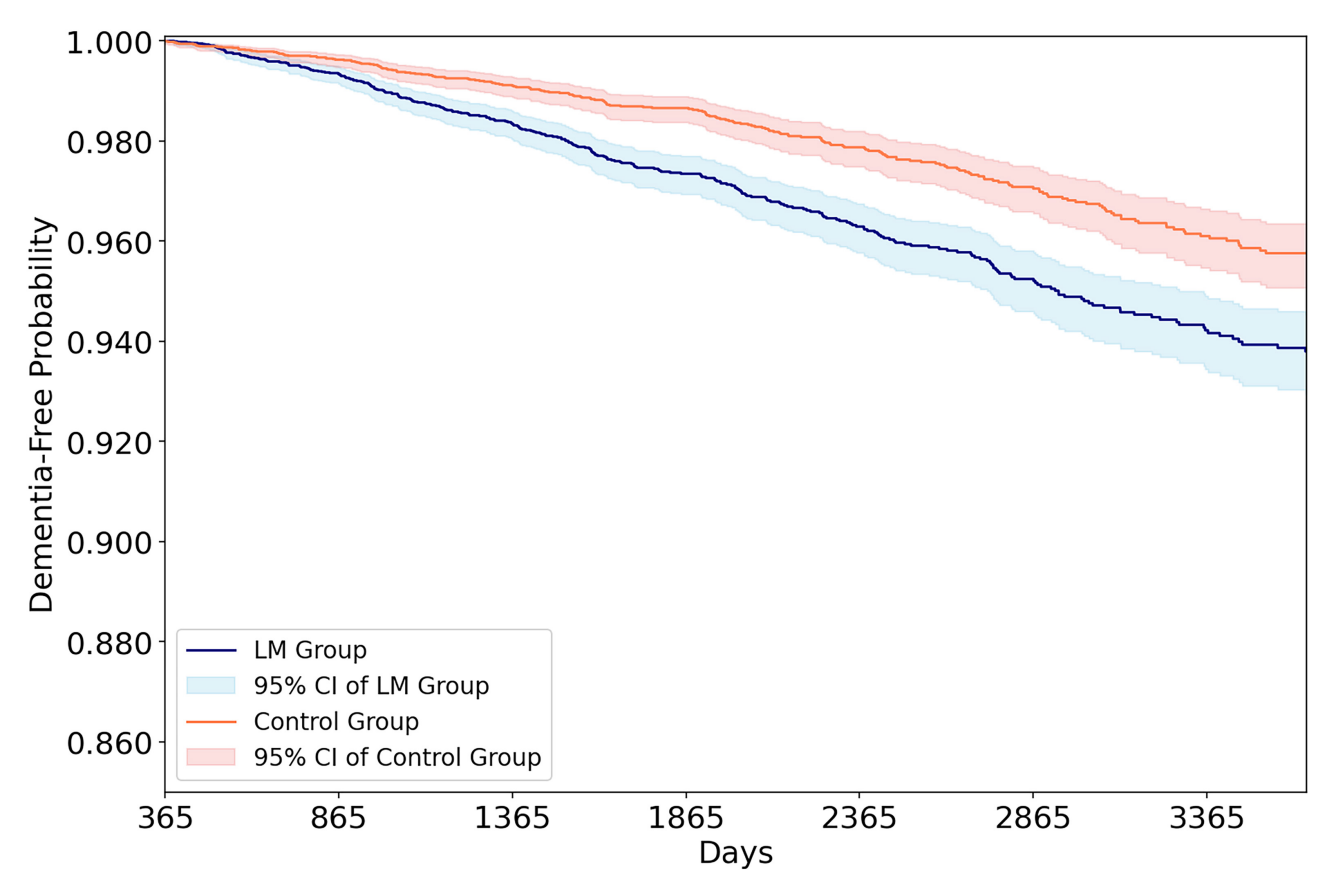
Kaplan–Meier curves for dementia-free survival in patients with low magnesium status vs. normal magnesium status after propensity score matching. The outcome assessment window began after a 1-year landmark period. The shaded area represents a 95% confidence interval. Patients with low magnesium levels had significantly lower dementia-free survival than matched controls over the 10-year follow-up period (hazard ratio: 1.62, confidence interval: 1.36–1.94, log-rank *p* < 0.001). LM: low magnesium.

Over the 10-year follow-up window, the cumulative incidence of newly diagnosed dementia was higher in the low magnesium cohort than in the matched reference cohort (2.53% vs. 1.78%; HR 1.62, 95% CI 1.36–1.94, *p* < 0.001; Table 2, Figure 1). The corresponding E-value was 2.62 for the point estimate and 2.06 for the lower confidence bound. For the prespecified secondary outcomes, higher event rates were observed in the low magnesium cohort for vascular dementia (HR 1.97, *p* = 0.001), other types of dementia (HR 1.63, *p* < 0.001), cognitive dysfunction (HR 1.43, *p* = 0.009), and all-cause mortality (HR 1.29, *p* < 0.001), whereas the association with Alzheimer's disease did not reach statistical significance (HR 1.43, *p* = 0.075).

**Table 2:** Association between low magnesium status and the 10-year risk of incident dementia and secondary outcomes.

Outcome	Low Mg group (*n* = 11,716) Events (%)	Control group (*n* = 11,716) Events (%)	HR (95% CI)	*p* value
Primary outcome				
Overall dementia	296 (2.53)	208 (1.78)	1.62 (1.36–1.94)	< 0.001
Secondary outcomes				
Alzheimer's disease	55 (0.47)	44 (0.38)	1.43 (0.96–2.13)	0.075
Vascular dementia	59 (0.50)	34 (0.29)	1.97 (1.29–3.00)	0.001
Other type of dementia	266 (2.27)	187 (1.60)	1.63 (1.35–1.96)	< 0.001
Cognitive dysfunction	122 (1.04)	97 (0.83)	1.43 (1.09–1.86)	0.009
Mortality	2,396 (20.45)	2,091 (17.85)	1.29 (1.22–1.37)	< 0.001
Positive control outcome				
Serum calcium < 8.5 mg/dL	6,035 (51.51)	5,661 (48.32)	1.30 (1.26–1.35)	< 0.001
Negative control outcome				
Appendicitis	50 (0.43)	63 (0.54)	0.89 (0.61–1.28)	0.519
Healthcare utilization validation				
Healthcare visit	9,738 (83.12)	10,108 (86.28)	1.02 (0.99–1.05)	0.135
Exposure validation				
Subsequent low magnesium	6,184 (52.78)	3,591 (30.65)	2.55 (2.44–2.66)	< 0.001

Mg, magnesium; HR, hazard ratio; CI, confidence interval.

The positive control outcome (hypocalcemia) was more frequent in the low magnesium cohort (HR 1.30, *p* < 0.001), while the negative control outcome of appendicitis showed no significant between-group difference (HR 0.89, *p* = 0.519). Healthcare visit frequency was comparable between the cohorts (HR 1.02, *p* = 0.135), and subsequent low magnesium events occurred more frequently in the exposure cohort (HR 2.55, *p* < 0.001; Table 2). In the 5-year analysis, the association with overall dementia was of greater magnitude than that at 10 years (HR 1.94, *p* < 0.001), with consistent patterns for vascular dementia (HR 2.19, *p* = 0.004), other dementia types (HR 1.95, *p* < 0.001), and mortality (HR 1.28, *p* < 0.001); associations with Alzheimer's disease (*p* = 0.19) and cognitive dysfunction (*p* = 0.096) did not reach statistical significance at 5 years (Supplementary Table 2).

### 3.3 Sensitivity analyses

The findings were broadly consistent across the three prespecified sensitivity analyses (Table 3). After excluding patients who died during follow-up (Model I), the association with overall dementia persisted (HR 1.32, *p* = 0.006). Restricting patients with more than one healthcare encounter (Model II) yielded similar results (HR 1.37, *p* < 0.001), as did restricting patients older than 65 years (Model III; HR 1.62, *p* < 0.001). Associations with overall dementia, other types of dementia, and mortality reached statistical significance in all applicable sensitivity models, whereas associations with Alzheimer's disease, vascular dementia, and cognitive dysfunction reached statistical significance in some, but not all, models.

**Table 3:** Sensitivity analyses of the association between low magnesium status and the 10-year risk of incident dementia.

Outcomes	Model I (*n* = 8,057 for each group)		Model II (*n* = 9,434 for each group)		Model III (*n* = 7,734 for each group)	
	HR (95% CI)	*P* value	HR (95% CI)	*P* value	HR (95% CI)	*P* value
Overall dementia	1.32 (1.08–1.61)	0.006	1.37 (1.15–1.63)	< 0.001	1.62 (1.34–1.94)	< 0.001
Alzheimer's disease	1.08 (0.71–1.65)	0.722	1.23 (0.84–1.81)	0.290	1.80 (1.18–2.74)	0.010
Vascular dementia	1.60 (0.99–2.60)	0.053	1.57 (1.05–2.35)	0.028	1.76 (1.15–2.70)	0.010
Other type of dementia	1.30 (1.06–1.60)	0.013	1.37 (1.14–1.64)	0.001	1.62 (1.34–1.97)	< 0.001
Cognitive dysfunction	1.64 (1.22–2.22)	0.001	1.36 (1.04–1.77)	0.025	1.28 (0.95–1.73)	0.110
Mortality	–	–	1.27 (1.19–1.35)	< 0.001	1.30 (1.21–1.39)	< 0.001

Model I excluded patients who died during follow-up; Model II included only patients with more than one healthcare encounter; and Model III was restricted to patients aged > 65 years. HR, hazard ratio; CI, confidence interval.

### 3.4 Subgroup analyses

In subgroup analyses by sex, the associations between low magnesium and overall dementia were comparable in men (HR 1.46, *p* = 0.004) and women (HR 1.35, *p* = 0.009), with no evidence of effect modification (*P* for interaction = 0.663; Table 4). When stratified by TBI severity, the associations with overall dementia were of similar magnitude in mild (HR 1.46, *p* = 0.003) and moderate-to-severe TBI (HR 1.46, *p* < 0.001), with no significant interaction (*P* for interaction = 1.000; Table 5). No statistically significant heterogeneity was detected across the interaction tests for the remaining outcomes.

**Table 4:** Subgroup analysis of the association between low magnesium status and dementia risk stratified by sex.

Outcomes	Male		Female		*P* for interaction
	HR (95% CI)	*P* value	HR (95% CI)	*P* value	
Overall dementia	1.46 (1.13–1.90)	0.004	1.35 (1.08–1.70)	0.009	0.663
Alzheimer's disease	1.22 (0.63–2.37)	0.552	1.36 (0.84–2.19)	0.208	0.803
Vascular dementia	2.53 (1.34–4.75)	0.003	1.17 (0.68–2.00)	0.574	0.145
Other type of dementia	1.39 (1.07–1.82)	0.015	1.41 (1.11–1.80)	0.005	0.939
Cognitive dysfunction	1.51 (1.01–2.26)	0.042	1.41 (0.99–2.01)	0.057	0.808
Mortality	1.25 (1.16–1.35)	< 0.001	1.27 (1.17–1.39)	< 0.001	0.787

HR, hazard ratio; CI, confidence interval.

**Table 5:** Subgroup analysis of the association between low magnesium status and dementia risk stratified by TBI severity.

Outcomes	Mild TBI		Moderate to severe TBI		*P* for interaction
	HR (95% CI)	*P* value	HR (95% CI)	*P* value	
Overall dementia	1.46 (1.13–1.87)	0.003	1.46 (1.21–1.78)	< 0.001	1.000
Alzheimer's disease	1.61 (0.92–2.82)	0.089	1.60 (0.98–2.60)	0.058	0.988
Vascular dementia	1.00 (0.55–1.84)	0.988	1.77 (1.13–2.76)	0.011	0.147
Other type of dementia	1.56 (1.20–2.03)	0.001	1.49 (1.22–1.82)	< 0.001	0.789
Cognitive dysfunction	1.46 (1.00–2.11)	0.046	1.47 (1.10–1.96)	0.008	0.978
Mortality	1.38 (1.25–1.52)	< 0.001	1.27 (1.19–1.35)	< 0.001	0.170

Mild TBI was defined as a concussion (ICD-10-CM: S06.0). Moderate-to-severe TBI was defined as intracranial injury involving structural brain lesions, including traumatic subdural hemorrhage (S06.5), traumatic subarachnoid hemorrhage (S06.6), epidural hemorrhage (S06.4), and other intracranial injuries under S06, excluding isolated concussion. HR, hazard ratio; CI, confidence interval; TBI, traumatic brain injury.

### 3.5 Adjusted hazard ratios from multivariable cox regression

In the multivariable Cox model incorporating demographic factors, comorbidities, and TBI subtypes, low magnesium status remained associated with incident dementia (adjusted HR 1.50, 95% CI 1.33–1.70, *p* < 0.001; Table 6). Older age, diabetes mellitus, mood disorders, alcohol-related disorders, cerebrovascular diseases, chronic kidney disease, and traumatic subdural hemorrhage were independently associated with incident dementia.

**Table 6:** Multivariable Cox proportional hazards regression for incident dementia (low magnesium status vs. normal magnesium status).

Variable	HR (95% CI)	*P*-value
Low Mg vs. control	1.50 (1.33–1.70)	< 0.001
Male	0.95 (0.85–1.06)	0.371
Age at index	1.09 (1.08–1.09)	< 0.001
Essential hypertension	1.08 (0.95–1.22)	0.247
Diabetes mellitus	1.23 (1.08–1.39)	0.001
Nicotine dependence	1.06 (0.90–1.26)	0.497
Mood disorders	1.44 (1.26–1.64)	< 0.001
Alcohol-related disorders	1.31 (1.08–1.59)	0.006
Cerebrovascular diseases	1.28 (1.12–1.46)	< 0.001
Diseases of liver	1.15 (0.97–1.37)	0.112
Overweight and obesity	0.97 (0.83–1.14)	0.712
Disorders of thyroid gland	1.02 (0.90–1.17)	0.729
Chronic kidney disease	1.25 (1.07–1.47)	0.007
Malnutrition	1.12 (0.88–1.44)	0.358
Traumatic subarachnoid hemorrhage	1.10 (0.90–1.35)	0.368
Traumatic subdural hemorrhage	1.20 (1.02–1.40)	0.024

HR, hazard ratio; CI, confidence interval; Mg: magnesium.

### 3.6 High magnesium status and dementia risk in patients with TBI

In the parallel analysis comparing 10,675 patients with high magnesium levels against the same matched reference cohort, an association with overall dementia was also observed (HR 1.57, *p* < 0.001; Supplementary Table 3). Higher event rates were also noted for Alzheimer's disease (HR 1.96, *p* = 0.002), vascular dementia (HR 2.06, *p* = 0.001), other dementia types (HR 1.55, *p* < 0.001), cognitive dysfunction (HR 1.65, *p* < 0.001), and mortality (HR 1.15, *p* < 0.001). The positive and negative control outcomes behaved as expected, and persistence of the exposure was confirmed by subsequent high-magnesium events (HR 2.17, *p* < 0.001). In the multivariable Cox regression analysis, high magnesium status remained independently associated with incident dementia (adjusted HR 1.51, *p* < 0.001; Supplementary Table 4).

## 4 Discussion

To our knowledge, no previous large-scale study has specifically evaluated whether serum magnesium abnormalities are associated with the risk of long-term dementia in adults with TBI. In this cohort, both low and high serum magnesium levels were associated with a higher 10-year risk of incident dementia, suggesting that deviations in either direction may reflect or contribute to an unfavorable neurobiological milieu following TBI. Furthermore, the principal findings remained consistent across sensitivity analyses and subgroups defined by sex and TBI severity. The association appeared stronger at 5 years than at 10 years, a temporal pattern that may partly reflect changes in magnesium status over an extended follow-up. These findings extend the U-shaped association between magnesium status and dementia beyond the general population to a clinically distinct TBI cohort, highlighting the potential relevance of disrupted magnesium homeostasis in shaping long-term neurodegenerative vulnerability after brain injury.

Several population-based cohort studies have examined the association between serum magnesium levels and dementia risk in the general adult population. The Rotterdam Study, which followed 9,569 participants over a median of 7.8 years, reported that both low ( ≤ 0.79 mmol/L) and high (≥0.90 mmol/L) serum magnesium levels were associated with an approximately 30% increased risk of dementia (23). The ARIC study, which included 12,040 participants with a median follow-up of 24.2 years, reported that low midlife serum magnesium levels were associated with a 24% higher risk of incident dementia (21). The effect sizes observed in our TBI cohort were notably larger than those reported in these general populations, consistent with the possibility that pre-existing brain vulnerability may modify this association. Importantly, both the Rotterdam and ARIC studies (21, 23) relied on single baseline magnesium measurements, whereas our study required two qualifying measurements within 1 year, thereby capturing a more persistent exposure pattern and reducing the risk of misclassification. Moreover, a propensity score model was deliberately constructed to account for the clinical complexity of the TBI population. In addition to demographic and cardiometabolic factors, matching incorporated TBI subtypes, vitamin D deficiency (37), nutritional indicators, and medication classes (38–41) relevant to cognitive outcomes.

In the primary analysis of low magnesium, the association with vascular dementia was the strongest among all subtypes (HR 1.97), followed by other dementia types (HR 1.63), whereas the association with Alzheimer's disease did not reach statistical significance (HR 1.43, *p* = 0.075). The prominence of vascular dementia is biologically plausible. When serum hypomagnesemia reflects reduced central nervous system magnesium availability, diminished voltage-dependent blockade of N-methyl-D-aspartate receptors may increase calcium influx and excitotoxic injury (13, 14, 42). Intracellular magnesium depletion has also been demonstrated after experimental TBI (43). Such depletion may impair Mg–ATP-dependent energy metabolism and mitochondrial homeostasis, thereby increasing oxidative stress (11–14, 19, 44). In addition, magnesium deficiency may promote neuroinflammation and compromise endothelial and blood–brain barrier integrity (45–48). These processes may collectively contribute to cerebral small-vessel injury, neurodegeneration, and impaired synaptic plasticity after TBI. Although direct comparison with prior studies is limited, the Rotterdam Study (23) also found that the association between serum magnesium and Alzheimer's disease was directionally similar to that for overall dementia but did not reach statistical significance, broadly aligning with the weaker Alzheimer's-specific association observed in our low-magnesium analysis. However, when the analysis was restricted to patients older than 65 years (sensitivity analysis Model III), all dementia subtypes, including Alzheimer's disease (HR 1.80, *p* = 0.010), showed significant associations with low magnesium, suggesting that age may be an important modifier of subtype-specific vulnerability.

Interestingly, high magnesium levels were associated with a higher risk of incident Alzheimer's disease. This divergence suggests that the two extremes of magnesium dysregulation may not confer dementia risk through symmetrical mechanisms. Low magnesium may directly amplify excitotoxic, mitochondrial, inflammatory, and vascular injury. Although population-based studies have reported an increased dementia risk at both low and high circulating magnesium concentrations (23, 25), the biological interpretation of high serum magnesium remains uncertain. High serum magnesium may reflect reduced renal excretion or exogenous magnesium exposure (49, 50), and circulating magnesium concentrations do not necessarily correspond to intracellular magnesium status (51). Experimental elevation of brain magnesium has been shown to upregulate NR2B-containing NMDA receptor signaling, enhance synaptic plasticity, and improve learning and memory in rats (52). Therefore, the observed association with high serum magnesium should not be interpreted as evidence of direct magnesium neurotoxicity. Biomarker-confirmed dementia phenotyping is needed to determine whether high magnesium levels are linked to Alzheimer's-specific pathobiology, reflect underlying comorbidities, or represent a distinct marker of neurodegenerative vulnerability.

Low magnesium levels were associated with a similar increase in overall dementia risk after mild and moderate-to-severe TBI, with no evidence of effect modification by injury severity. This pattern contrasts with prior epidemiological evidence of a severity-dependent gradient in TBI-associated dementia risk (53, 54). The absence of such a gradient suggests that magnesium dysregulation may contribute to post-TBI cognitive vulnerability through mechanisms that are not primarily governed by the initial mechanical injury. Because no prior study has examined whether a modifiable metabolic factor differentially influences dementia risk across TBI severity strata, a direct comparison with the existing literature remains limited.

Several analytical safeguards were incorporated to support the internal validity of these findings. The positive control outcome of hypocalcemia occurred more frequently in the low magnesium cohort, consistent with the established physiological coupling between magnesium and calcium homeostasis through parathyroid hormone regulation (55), whereas the negative control outcome of acute appendicitis showed no between-group difference. Healthcare visit frequency was comparable between the cohorts, arguing against differential surveillance as an explanation for the observed associations. The stability of the exposure definition was supported by the markedly higher recurrence of low magnesium levels within the exposure cohort, indicating that these patients consistently exhibited hypomagnesemia over time. This pattern suggests that the exposure classification reflects a sustained biological state rather than an isolated or transient laboratory variation. The E-value for the primary outcome was 2.62, indicating that an unmeasured confounder would need to be associated with both magnesium status and dementia by a risk ratio exceeding that of most known dementia risk factors to explain the observed association.

This study had several strengths. First, exposure was defined using repeated serum magnesium measurements, reducing misclassification relative to prior studies that were based on a single baseline value. Second, the 1-year landmark design strengthened temporal ordering by excluding early dementia diagnoses that may have reflected a pre-existing cognitive decline. Third, dementia was examined not only as a composite endpoint but also by subtype, allowing the identification of distinct patterns for Alzheimer's disease, vascular dementia, and other types of dementia. Finally, the parallel evaluation of low and high magnesium levels within the same study design allowed the two extremes of magnesium status to be interpreted against a common comparator, thereby strengthening the evidence for a U-shaped association with dementia risk.

Several limitations should be acknowledged. First, the observational design precludes causal inference; the observed associations may reflect residual confounding by unmeasured factors, such as dietary patterns, socioeconomic status, or genetic susceptibility. Second, serum magnesium represents less than 1% of total body magnesium and may not accurately reflect intracellular or central nervous system magnesium status (56), particularly in TBI, where blood–brain barrier integrity may be compromised. In addition, although requiring two qualifying magnesium measurements within 1 year reduced the likelihood of classifying patients on the basis of a single transient value, this criterion cannot fully establish chronic magnesium homeostasis. The interval between measurements could vary within the 1-year window, and serum magnesium may still reflect recent nutritional, clinical, renal, or treatment-related status. Residual exposure misclassification is therefore possible. Third, TBI severity was classified using ICD-10-CM diagnostic codes rather than clinical scales, such as the Glasgow Coma Scale, which may result in misclassification of injury severity and limit the precision of subgroup analyses stratified by TBI severity. Additionally, the present study assessed only TBI severity and did not account for the number or recurrence of TBI episodes, which is a recognized independent risk factor for long-term neurodegeneration and may further modify the association between magnesium and dementia. Fourth, dementia outcomes were ascertained using diagnostic codes rather than standardized cognitive assessments, potentially underestimating the true incidence. Fifth, although magnesium supplementation was balanced between cohorts after matching (approximately 33%), it remains possible that patients who maintained low levels despite supplementation represented a phenotype of more severe depletion, which could influence the observed associations. Finally, the TriNetX network predominantly comprises healthcare organizations in the United States, which may limit the generalizability of the findings to other populations.

## 5 Conclusion

In this large propensity score–matched cohort of adults with prior TBI, both low and high serum magnesium levels were associated with a greater 10-year dementia risk. The associations remained consistent across sensitivity analyses, subgroup analyses, and multivariable adjustments and were supported by the expected positive and negative control outcome patterns. These findings suggest that serum magnesium levels may serve as a clinically accessible indicator of long-term cognitive risk after TBI. However, prospective studies are needed to determine whether monitoring or modifying magnesium levels impacts post-traumatic dementia trajectories.

## Data Availability

The datasets presented in this article are not readily available because the data were obtained from the TriNetX Global Collaborative Network under institutional license and are not publicly available. Restrictions apply because the dataset is proprietary and consists of de-identified electronic health record data from participating healthcare organizations. The authors cannot share or export individual-level data; access may be requested directly from TriNetX under an appropriate data-use agreement. Requests to access the datasets should be directed to https://live.trinetx.com.
