# Supplementary material for: U-shaped pattern in the association between serum magnesium levels and long-term dementia risk after traumatic brain injury: a retrospective cohort study
Source: Front Nutr. 2026 Aug 7;13:1870929. doi: 10.3389/fnut.2026.1870929 (PMC13495782; doi:10.3389/fnut.2026.1870929)
Supplement: Supplementary file 1 [file Table_1.docx]

**Supplementary Table 1. Variables included in propensity score matching and corresponding codes**

| Category | Variable | Code (ICD-10-CM / ATC / LOINC) |
| --- | --- | --- |
| Demographics | Age at index | Continuous |
|  | Sex | Male / Female |
|  | Race/ethnicity | White (2106-3), Black (2054-5), Asian (2028-9) |
| Cardiometabolic comorbidities | Hypertension | I10 |
|  | Diabetes mellitus | E08–E13 |
|  | Obesity / overweight | E66 |
|  | Ischemic heart disease | I20–I25 |
|  | Heart failure | I50 |
|  | Atrial fibrillation | I48 |
| Lifestyle-related conditions | Nicotine dependence | F17 |
|  | Alcohol-related disorders | F10 |
| Neurological and psychiatric conditions | Mood disorders | F30–F39 |
|  | Anxiety disorders | F40–F48 |
|  | Sleep disorders | G47 |
|  | Epilepsy | G40 |
| Systemic and chronic conditions | Chronic kidney disease | N18 |
|  | Liver disease | K70–K77 |
|  | COPD | J44 |
|  | Thyroid disorders | E00–E07 |
|  | Malnutrition | E40–E46 |
|  | Systemic connective tissue disease | M30–M36 |
|  | Cerebrovascular disease | I60–I69 |
| Injury-related variables (TBI subtypes) | Concussion (mild TBI) | S06.0 |
|  | Epidural hemorrhage | S06.4 |
|  | Subdural hemorrhage | S06.5 |
|  | Subarachnoid hemorrhage | S06.6 |
| Nutritional/metabolic factors | Vitamin D deficiency | E55 |
| Medication exposures | Benzodiazepines | CN302 |
|  | Anticonvulsants | CN400 |
|  | Anticholinergics | S01FA |
|  | Central nervous system medications | CN000 |
|  | Diuretics | CV700 |
|  | Insulins and analogues | A10A |
|  | Blood glucose–lowering drugs (non-insulin) | A10B |
|  | Magnesium supplementation | TN460 |
| Laboratory variables | Serum magnesium | LOINC: 19123-9 |
|  | Hemoglobin | LOINC: 9014 |
|  | HbA1c | LOINC: 9037 |
|  | C-reactive protein | LOINC: 9063 |
|  | Albumin | LOINC: 9045 |
|  | eGFR | LOINC: 98979-8 |
|  | Vitamin B12 | LOINC: 9065 |
|  | Body mass index | LOINC: 9083 |

**Supplementary Table 2. Association between low magnesium status and the 5-year risk of incident dementia and secondary outcomes.**

| Outcome | Low Mg group  ( n = 11,716) | Control group  (n = 11,716) | HR (95% CI) | p value |
| --- | --- | --- | --- | --- |
|  | Events (%) | Events (%) |  |  |
| Primary outcome |  |  |  |  |
| Overall dementia | 189 (1.61) | 107 (0.91) | 1.94 (1.53–2.46) | <0.001 |
| Secondary outcomes |  |  |  |  |
| Alzheimer’s disease | 30 (0.26) | 23 (0.20) | 1.44 (0.83–2.47) | 0.190 |
| Vascular dementia | 38 (0.32) | 19 (0.16) | 2.19 (1.26–3.79) | 0.004 |
| Other type of dementia | 165 (1.41) | 93 (0.79) | 1.95 (1.51–2.52) | <0.001 |
| Cognitive dysfunction | 76 (0.65) | 63 (0.54) | 1.33 (0.95–1.85) | 0.096 |
| Mortality | 1,662 (14.19) | 1,414 (12.07) | 1.28 (1.20–1.38) | <0.001 |

Mg, magnesium; HR, hazard ratio; CI, confidence interval.

**Supplementary Table 3. Association between high magnesium status and the 10-year risk of incident dementia, secondary outcomes, and validation measures**

| Outcome | High Mg group  (n = 10,675) | Control group  (n = 10,675) | HR (95% CI) | p value |
| --- | --- | --- | --- | --- |
|  | Events (%) | Events (%) |  |  |
| Overall dementia | 279 (2.61) | 201 (1.88) | 1.57 (1.31–1.88) | <0.001 |
| Alzheimer’s disease | 54 (0.51) | 31 (0.29) | 1.96 (1.26–3.05) | 0.002 |
| Vascular dementia | 55 (0.52) | 30 (0.28) | 2.06 (1.32–3.21) | 0.001 |
| Other type of dementia | 251 (2.35) | 183 (1.71) | 1.55 (1.28–1.87) | <0.001 |
| Cognitive dysfunction | 136 (1.27) | 93 (0.87) | 1.65 (1.26–2.14) | <0.001 |
| Mortality | 2,046 (19.17) | 1,982 (18.57) | 1.15 (1.09–1.23) | <0.001 |
| Serum calcium <8.5 mg/dL | 4,681 (43.85) | 4,925 (46.14) | 1.08 (1.04–1.13) | <0.001 |
| Appendicitis | 46 (0.43) | 47 (0.44) | 1.09 (0.73–1.64) | 0.68 |
| Healthcare visit | 8,353 (78.25) | 9,040 (84.68) | 0.99 (0.96–1.02) | 0.471 |
| Subsequent high Magnesium | 4,725 (44.26) | 3,045 (28.53) | 2.17 (2.07–2.27) | <0.001 |

HR, hazard ratio; CI, confidence interval; Mg: magnesium

**Supplementary Table 4.Multivariable Cox proportional hazards regression for incident dementia (high magnesium status vs normal magnesium status).**

| Variable | HR(95% CI) | P-value |
| --- | --- | --- |
| High Mg vs. control | 1.51 (1.34–1.71) | <0.001 |
| Male | 0.94 (0.84–1.05) | 0.292 |
| Age at index | 1.09 (1.08–1.09) | <0.001 |
| Essential hypertension | 1.12 (0.98–1.27) | 0.088 |
| Diabetes mellitus | 1.25 (1.10–1.42) | <0.001 |
| Nicotine dependence | 1.08 (0.91–1.29) | 0.365 |
| Mood disorders | 1.43 (1.25–1.63) | <0.001 |
| Alcohol-related disorders | 1.33 (1.09–1.63) | 0.005 |
| Cerebrovascular diseases | 1.25 (1.09–1.43) | 0.001 |
| Diseases of liver | 1.23 (1.03–1.47) | 0.022 |
| Overweight and obesity | 0.95 (0.81–1.11) | 0.515 |
| Disorders of thyroid gland | 1.03 (0.90–1.18) | 0.656 |
| Chronic kidney disease | 1.08 (0.92–1.27) | 0.365 |
| Malnutrition | 1.18 (0.93–1.49) | 0.179 |
| Traumatic subarachnoid hemorrhage | 1.06 (0.87–1.30) | 0.574 |
| Traumatic subdural hemorrhage | 1.22 (1.05–1.43) | 0.011 |

HR, hazard ratio; CI, confidence interval; Mg: magnesium
